# A Preliminary Study on the Abnormal Deaths and Work Burden of Chinese Physicians: A Mixed Method Analysis and Implications for Smart Hospital Management

**DOI:** 10.3389/fpubh.2021.803089

**Published:** 2022-01-04

**Authors:** Jun Liang, Yunfan He, Linye Fan, Mingfu Nuo, Dongxia Shen, Jie Xu, Xu Zheng, Tong Wang, Hui Qian, Jianbo Lei

**Affiliations:** ^1^Information Technology Center, School of Medicine, Second Affiliated Hospital, Zhejiang University, Hangzhou, China; ^2^School of Public Health, Zhejiang University, Hangzhou, China; ^3^Key Laboratory of Cancer Prevention and Intervention, China National Ministry of Education, School of Medicine, Zhejiang University, Hangzhou, China; ^4^Department of Obstetrics and Gynecology, The Affiliated Hospital of Southwest Medical University, Luzhou, China; ^5^Health Science Center, Institute of Medical Technology, Peking University, Beijing, China; ^6^Editorial Department of Journal of Practical Oncology, Second Affiliated Hospital, School of Medicine, Zhejiang University, Hangzhou, China; ^7^Center for Medical Informatics, Peking University Third Hospital, Beijing, China; ^8^School of Public Health, Jilin University, Changchun, China; ^9^College of Computer Science and Technology, Zhejiang University, Hangzhou, China; ^10^School of Medical Informatics and Engineering, Southwest Medical University, Luzhou, China

**Keywords:** public health policy, abnormal deaths, physicians, work burden, health institutions

## Abstract

**Background:** The population of Chinese physicians is frequently threatened by abnormal death, including death by overwork or homicide. This is not only a health problem, but also a social problem that has attracted the attention of both hospitals and the government.

**Objective:** This study aims to analyze the characteristics of abnormal death in physicians in Chinese hospitals from 2007 to 2020 and to investigate the relationship between abnormal death and physician workload, in order to provide information for policy makers and request improvement technologies.

**Methods:** A mixed research method was used. In order to ensure accuracy and completeness, a relatively comprehensive search was conducted using multiple heterogeneous data sources on the abnormal death of physicians in Chinese hospitals from 2007 to 2020. The collected cases were then descriptively analyzed using the work-related overwork death risk concept framework and the deductive grounded theory approach. In addition, the workload of physicians was calculated between 2007 and 2019 based on three important workload indicators.

**Results:** Between 2007 and 2020, 207 abnormal death events of physicians on the Chinese mainland were publicly reported. Among the 207 victims, the majority (~79%) died from overwork or sudden death. The number of victims who were men was 5.5 times higher than that of women, and victims were between the ages of 31–50 years. These physicians mainly belonged to the departments of surgery, anesthesiology, internal medicine, and orthopedics. Further analysis of the direct causes of death in cases of overwork death showed that 51 physicians (31.1%) died from cardiogenic diseases. Additionally, the per capita workload of physicians in China increased drastically by about 42% from 2007 to 2019, far exceeding physician workloads in Europe, Asia, and Australia (number of inpatients per physician in 2017: 72 vs. 55, 50, 45). The analysis revealed that there was a strong correlation between the number of abnormal deaths of physicians in China and the number of inpatients per physician (*r* = 0.683, *P* = 0.01).

**Conclusion:** High-intensity working conditions may be positively correlated with the number of abnormal deaths among physicians. Smart hospital technologies have the potential to alleviate this situation.

## Introduction

There is no doubt that physicians are among the most important professionals to the survival of the world's populations and therefore need protection in society. However, physicians worldwide continuously face growing pressure, especially in the Chinese context. In 2015, Shatkin analyzed data from the U.S. Bureau of Labor Statistics as well as O^*^NET, a job information website, and listed the top 27 high-pressure jobs out of 767 occupations in the United States. Of these, 11 of the top 27 high-pressure jobs were related to the medical industry and included obstetricians and gynecologists, surgeons, physicians, and stomatologists (ranked 5th, 6th, 15th, and 20th, respectively) (1). Similarly, the *National Physician Burnout* & *Suicide Report 2020: The Generational Divide*, published by Medscape in 2020, surveyed 15,000 doctors in 29 clinical departments. The results showed that 42% of surveyed doctors reported being overstrained at work, and the ratio of overworked doctors was more than 40% in most departments (2). In the UK, the *Medscape UK Doctors' Burnout* & *Life Style Survey*, which was also published by Medscape in 2020, surveyed 1,082 doctors and found that ~37% were very tired at work (3). According to the survey results of the *White Paper on the Occupational Status of Doctors in China*, which was published by the Chinese Medical Association in 2017 and surveyed 146,200 doctors, the overall level of psychological exhaustion in doctors was significantly higher than that of enterprise employees. In total, 51.3% of interviewees had psychological exhaustion above level 6 (severe), and the level of psychological exhaustion reached 69%.

One of the main goals of smart hospitals is to reduce the unnecessary workload of medical staff and return medical staff to patients. The “smart hospital” strategy is one of the most important policies issued by the Chinese government for the medical industry in recent years. The scope of smart hospitals is mainly divided into three areas. The first, “smart medical care” for medical staff (4, 5), focuses on the construction of interoperability between the various heterogeneous systems and the hospital's healthcare information technology (HIT) with electronic health records (EHRs) as the core, so as to provide patients with complete information support when visiting a doctor. The second, “smart management” for hospital administrators (6), is based on the refined cost accounting of HIT and the fine-grained management of internal logistics, administration, and equipment of the hospital. Third, “smart service” for patients (7) includes self-hosting, patient portal, Internet hospitals, and telemedicine services deployed in hospitals, which enables patients to experience more convenient and faster self-service as well as remote services.

The health and living conditions of physicians are of paramount importance to medical care, but excessive work pressure may impact their health or even lead to death by overwork. The first documented case of death by overwork occurred in Japan in 1969 (8). Uehata of the Institute of Public Health in Japan, reported five possible work patterns that may lead to overwork death: all-weather, high-intensity work; extremely long working hours or long-term, day-night reversal; continuous work without rest; extremely heavy physical work; and work with high mental pressure (9). Unfortunately, some of these elements have become mainstream for many physicians in China. In addition, physicians in China are frequently threatened by abnormal death, including death by overwork or homicide. In 2010, there were a total of 17,000 cases of patients or their families physically beating physicians in China, while in 2013, such events rose to more than 70,000 cases (10). The detailed information can be found in Appendix 1, which includes a complete description of the work status and working environment of physicians in China.

Describing the current status of abnormal death among physicians, assessing their prevalence, analyzing their characteristics, and exploring their relationship with workload are effective ways to help policy makers and hospital managers understand physicians' work and health status. In turn, this can support the subsequent establishment of more effective prevention, intervention, and improvement strategies. However, in terms of research content, this study primarily focuses on physical, chemical, and biological occupational exposure factors, while paying relatively little attention to social factors in hospital environments (11, 12). From the perspective of research objects, many studies on the health and psychological factors of physicians in China have focused on overwork death events without paying attention to other malignant deaths (13–15). Literature concerning the relationship between physicians' workload and the number of abnormal deaths is very rare. In terms of research methods, most of the studies are either based on literature databases or web pages with single information sources, and few studies have combined multiple data sources. We conducted a careful literature search using a set of key search terms and various combinations of search terms in an attempt to discover relevant research. This included exploring the Wanfang Data (16), China National Knowledge Infrastructure (17), and VIP Journal Integration Platform (18), which are three of the most commonly used Chinese databases. Search results showed that existing literature primarily focuses on the phenomena of physicians overworking or experiencing overwork death in China, but an analysis of other abnormal death cases and the impact of physician workload on perpetuating such phenomena is lacking.

In view of the above limitations, we collected data from various angles (including web pages, social media, and academic databases) in order to comprehensively explore the abnormal death phenomenon of physicians based on reports, news, and literature on abnormal deaths from 2007 to 2020, using a mixed method. Following this, we described and analyzed the current situation and summarized its general characteristics. In 2018, China was not only the largest developing country but also the most heavily populated country worldwide, yet it relied on only 3.6 million physicians to support the largest global healthcare service system (19). As China's healthcare system differs from that of other countries, some of the research results cannot be extrapolated to other contexts; however, due to the universality of healthcare services, healthcare systems worldwide have the phenomenon of insufficient medical resources, as well as the heavy work burden and great work pressure of physicians (20–22), albeit to varying degrees. Therefore, we believe that the opinions and results of this study have a certain degree of pervasiveness and universality. At the theoretical level, this study provides the latest status and insights for research on the overworked status of the Chinese physician population. To the best of our knowledge, there are few mixed studies focusing on the current status of physician workload and sudden death together. At the work level, it is helpful for policy makers and hospital managers to understand the work and health status of physicians who are the main executors of the “smart hospital” policy and to provide information for better implementation of the policy in practice.

## Materials and Methods

We adopted a mixed research approach that combined qualitative and quantitative analysis.

### Data Collection and Processing

First, data on abnormal deaths of physicians were collected through a comprehensive search on Internet sites and academic databases. Google and Baidu were used for the web search since Google is the world's top search engine, while Baidu is the Chinese search engine that ranks second globally (23). For social media, the Sogou search engine was used to extrapolate articles from official WeChat accounts. WeChat is the most widely used social media application in China and has the largest number of Chinese users (24), while Sogou is the fifth largest Chinese search engine worldwide and the only search engine that publicly supports searching WeChat social media (25). Regarding academic literature, the three largest Chinese literature databases (namely CNKI, VIP, and Wanfang) were used for Chinese literature searches, whereas PubMed and Web of science were used for the English literature search. The types of papers included in this study were Articles and Reviews, and the investigated period of time was from 2007 to 2020. To facilitate the search, a series of keywords and keyword combinations were established. We believe that these datasets already cover the content that needs to be searched. Specifically, two graduate students conducted the search and independently evaluated the content obtained from these three data sources. In addition, a graduate student conducted a random inspection of a subset of results. In case of differences and disagreements, they would be resolved through group discussions until a consensus was reached. Appendix 2 details the specific search terms used herein.

Second, the quantitative data of physicians' work were obtained from the 2007–2020 China Statistical Yearbook (26), which was issued by the Chinese National Bureau of Statistics. Specifically, three indicators were used, namely the number of registered physicians outpatients received per year, and inpatients received per year. On this basis, the average number of outpatients received by each physician per year, the average number of inpatients received by each doctor per year, and the average number of patients received by each physician per year were calculated to estimate the average workload of registered physicians in China.

### Data Analysis

In addition to conducting a descriptive analysis based on metadata (including information on the occurrence, demographic characteristics of the victim, department, and hospital level), we also used the work-related overwork death risk concept framework proposed by Uehata to encode overwork death cases based on the deductive grounded theory approach (27), and explored common patterns in the encoding results. The overwork death risk concept framework describes overwork death from four perspectives: lifestyle factors, physical conditions (i.e., specifically referring to underlying diseases), inducement events (i.e., specifically, whether physicians were continuously working overtime or taking the night shift), and medical causes of death (9). However, due to the lack of detailed information on the living habits and physical conditions of physicians who died by overworking, we focused on the last two factors. Using Pearson's product-moment correlation, we explored the relationship between the annual physician workload sequence and the corresponding abnormal death case sequence. Statistical significance was set at *p* < 0.05. The data were analyzed using IBM SPSS version 20 software (IBM Corp., Armonk, NY, USA).

## Results

### Descriptive Statistics

#### Relevant News, Cases, and Literature Review Results

Data retrieval was completed in December 2020. In terms of search engines, a total of 120,000 potentially related web pages were discovered through Baidu, while 2.91 million potentially related web pages were discovered through Google. In terms of social media, 10,663 WeChat official account articles on WeChat social media were retrieved through the Sogou engine. With reference to the verified search strategies and experience of our research team (28, 29), we focused on the relevant records recommended by three search engines (i.e., the top 753 search results recommended by Baidu, the top 996 search results recommended by Google, and the top 500 WeChat official account articles recommended by Sogou). From the literature databases, 287 related articles were found in Chinese and English.

**Figure 1:**
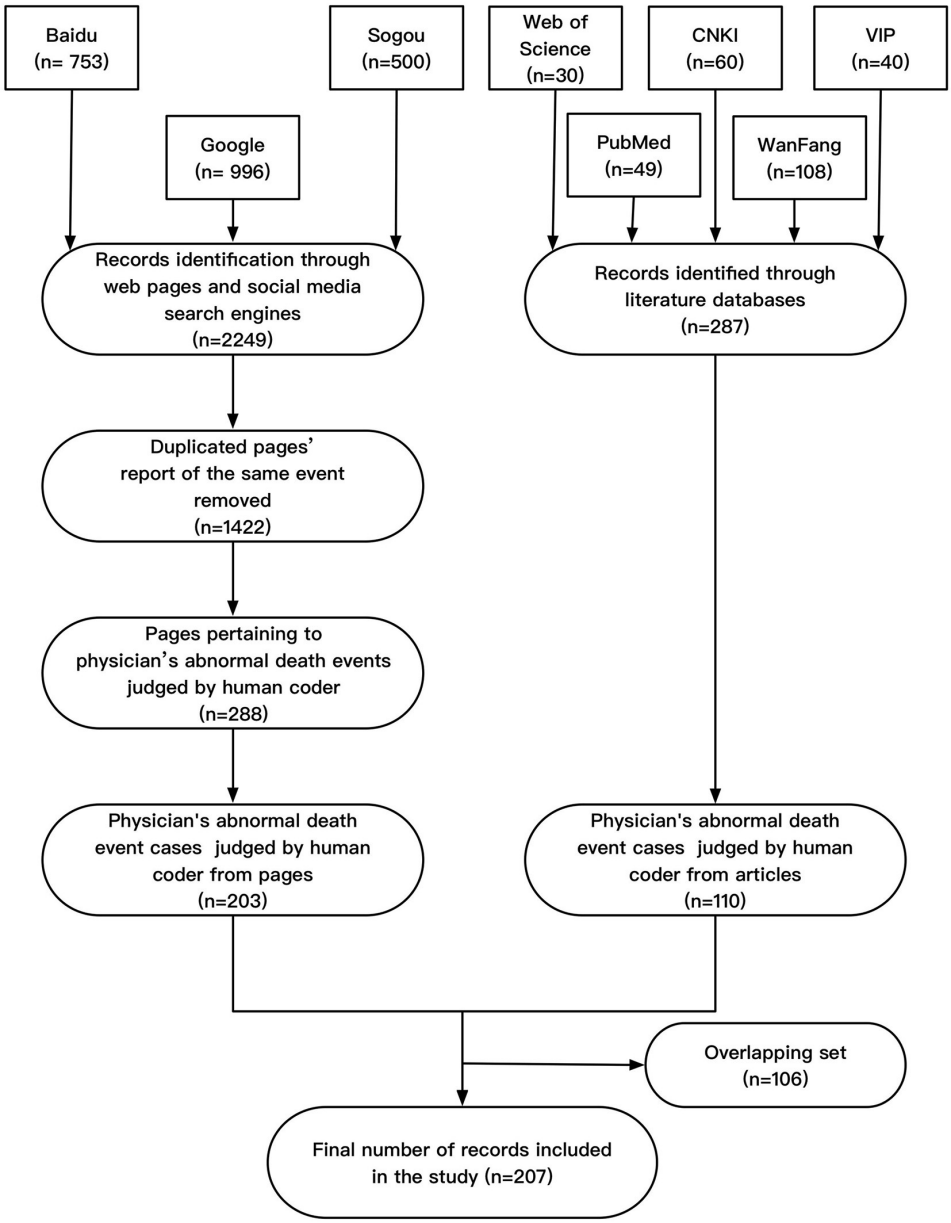
Flow chart of the data retrieval process.

The collected data were manually reviewed by two graduate students with a background in medical informatics, according to the established exclusion criteria. Exclusion criteria were as follows: (1) Non-physicians (those who were not licensed physicians, such as nurses and medical care-related civil servants) were included in the study; (2) Study participants did not die as a result of the incident (including serious injuries, minor injuries, recovery, etc.); (3) The occurrence time of the event was not between 2007 and 2020; (4) The time of the event or the victim's occupation could not be distinguished; and (5) The place of occurrence was not in China, or the victim was not a Chinese national. If any collected literature, reports, or news stories met any of the above exclusion criteria, they were excluded from the analysis. Following this process, 203 web pages and 110 articles were included in the analysis. The literature screening process and detailed flow chart are shown in Figure 1. In the specific context of this study, the performance of search engines and social media searches that were open to the public were significantly higher than those of literature databases. Among the 207 events identified, 203 were identified using search engines, whereas only 110 were identified using social media searches.

#### Sample Cases of Abnormal Deaths of Physicians

The descriptions of abnormal death among physicians in China from 2007 to 2020 were collected and summarized according to the methods shown in Table 1, and the specific case descriptions are detailed in Appendix 3. As shown in the table, abnormal deaths were mainly divided into three categories: overwork death, physician-patient disputes, and disputes with colleagues. Among them, overwork death accounted for the largest proportion (164 cases, 79.2%), followed by physician-patient disputes (36 cases, 7.4%). Based on the data, men were found to be about 5.5 times more likely to be victims of abnormal deaths than women. In terms of the level of medical institutions, tertiary hospitals had the highest number of cases, which was more than twice that of medical institutions at other levels.

**Table 1:** Descriptive statistics.

**Measurement index**	**Statistics**
Number of abnormal deaths	207
Time range	2007–2020
**Event classification**	
Overwork death	164
Deaths caused by physician-patient disputes	36
Deaths caused by colleague disputes	7
**Gender**	
Male	137
Female	26
**Hospital type +**	
Tertiary hospital	115
Secondary hospital	21
Primary hospital	10
Other	20
Provinces and cities with frequent abnormal deaths (10 or more)	Beijing (19), Jiangsu (18), Hubei (17), Zhejiang (15), Guangdong (14), Anhui (13), Shanghai (12), Henan (11), Hunan (10)
Departments with the most frequent abnormal deaths (10 or more)	Surgery (30), other departments (27)^*^, anesthesia (23), internal medicine (22), orthopedics (16)
**Professional ranking**	
Advanced	78
Intermediate	60
Primary	9
Not graded	20

* *Other departments include pharmacy, physical examination, emergency, prevention, and general practice*.

*+In China, hospitalization and emergency services are mainly provided by non-profit medical institutions, which are collectively referred to as “hospitals” and are divided into three levels. Tertiary hospitals are mainly responsible for the treatment of difficult and critical cases, clinical teaching, and scientific research, thereby corresponding to academic medical centers in the United States. Secondary hospitals are mainly responsible for the diagnosis and treatment of difficult and complicated diseases, common diseases, and frequently occurring diseases, thereby corresponding to general hospitals in the United States. Primary hospitals (primary medical institutions) are mainly engaged in prevention, rehabilitation, health care, nursing, and general outpatient services, thereby corresponding to community hospitals in the United States. Up to 2019, there were 11,266 primary hospitals, 9,805 secondary hospitals, and 2,779 tertiary hospitals (30)*.

It should be noted that the cumulative value of certain data in Table 1 is not equal to 207, because some of the victims' information (e.g., gender, age, job title, etc.) was not disclosed in some cases.

#### Trend Analysis

**Figure 2:**
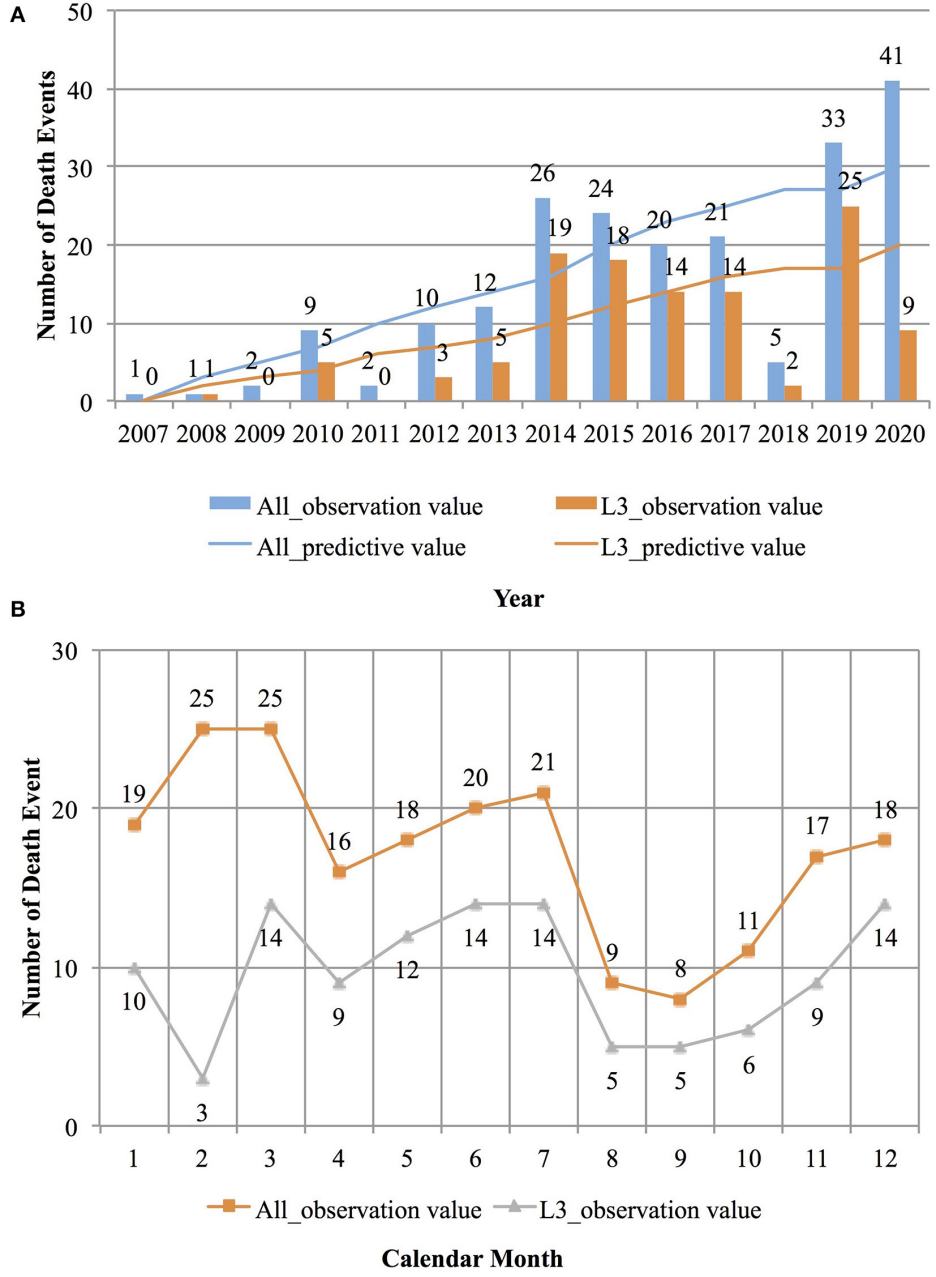
The distribution of abnormal deaths of doctors over time. **(A)** The distribution of the number of abnormal deaths of doctors by years. **(B)** The distribution of the number of abnormal deaths of doctors by months.

Figures 2A,B depict the distribution of the number of abnormal deaths of physicians by year and month, respectively. As shown in Figure 2A, from 2007 to 2020, the overall incidence of abnormal death among physicians showed an upward trend. The Holt-Winter time series model was used to fit the data, and the stationary R-square of the fitted model was *R*^2^ = 0.76. Based on the time series model, this number is expected to reach 45 by 2025. Further analysis of abnormal death cases of physicians in tertiary hospitals showed that a similar trend existed in this context. The Holt-Winter time series model was used to fit the data, and the stationary R-square of the fitted model was *R*^2^ = 0.74. Based on the time series model, this number is expected to reach 28 by 2025. This may only be a sliver of the full scope of the issue, because it can be assumed that only a few events were widely shared on the Internet. As shown in Figure 2B, the number of abnormal deaths was lowest in August and September. This may be because medical students are on summer vacation during this period and often have summer internships in several hospitals, which can help physicians carry out transactional auxiliary work. It is also possible that some abnormal deaths occurred during this period, but were not reported. In contrast, except for February, the proportion of abnormal deaths among physicians in tertiary hospitals exceeded 50%. It was further found that abnormal deaths in February mainly occurred in 2020, because many primary-level medical staff died of overwork while working on the frontlines of the COVID-19 pandemic.

### Analysis Based on the Overwork Death Victims' Analysis Framework

Tables 2, 3 describe the analysis results of 164 cases of overwork death among physicians based on the overwork death risk concept framework. The framework focuses on the direct cause of death, pre-death work status, lifestyle, and physical condition of victims of overwork death. However, due to the lack of detailed information on the living habits and physical conditions of physicians who died of overwork death, we focused on the first two factors.

**Table 2:** Direct causes of death in overwork death cases among physicians.

**Factor**	**Frequency**
Cardiac diseases (including acute myocardial infarction and acute heart failure)	51 (54.3%)
Suicide (falling from a building)	27 (28.7%)
Brain-derived diseases (including strokes and cerebral hemorrhages)	12 (12.8%)
Cancer	2 (2.1%)
Organ failure	2 (2.1%)

*It should be noted that the cumulative value of data in some cells is not equal to 207, because the cause of death or diagnosis was not disclosed in the reports of certain events*.

**Table 3:** Last work status of overwork death victims before death.

**Measurement index**	**Frequency (%)**
**Working continuously for more than 8 h**	
>8–12 h	64 (57.1)
13–24 h	20 (17.9)
25–48 h	24 (21.4)
>48 h	4 (3.6)

*It should be noted that the cumulative value of certain data in the Table is not equal to 207, because the final working status of doctors who died of overwork death was not disclosed in the reports of certain events*.

As shown in Table 2, among the 164 cases, 94 disclosed the direct cause of death. Most deaths (51 physicians) were caused by cardiogenic diseases, including acute myocardial infarction and acute heart failure. The second highest cause of death was suicide (27 physicians), due to world-weary behavior possibly caused by overwork, depression, and severe job burnout. The third cause was brain-derived diseases (12 physicians), including unexplained strokes and cerebral hemorrhages.

Table 3 shows the work status of the victims before death. Uehata believed that the work status of victims before death was a direct cause of overwork death (9). The 112-person report details physicians' work status before overwork death. Among them, about 64 physicians worked continuously for more than 8–12 h, 20 worked continuously for 13–24 h, 24 worked continuously for 25–48 h, and 4 physicians worked continuously for more than 2 days. Therefore, consistent with Uehata's research results, long working hours may prompt or even directly lead to overwork death.

### Workload of Physicians in China

During the period of 2007 to 2019, although the total number of physicians increased in China, the total workload increased faster, with the workload increasing about twice as fast as the number of physicians. While physicians in China increased by 85.7% (2.1 vs. 390 million), the number of outpatients per year increased by 163.6% (3.3 billion vs. 87 million) and the number of inpatients per year increased by 170.6% (98.3 vs. 2.66 million). In addition, the average number of outpatients received by each physician showed a stable trend since 2012, and even decreased by about 15.9% by 2019 (see Table 4 below for details).

**Table 4:** Number of professionally active physicians, outpatients, and inpatients in China from 2007 to 2019.

**Year**	**Physicians (million persons)**	**Outpatients (billion persons)**	**Inpatients (million persons)**	**Outpatients per physician (persons)**	**Inpatients per physician (persons)**
2007	2.1	3.3	98.3	1,571	47
2008	2.2	3.5	114.8	1,591	52
2009	2.3	5.5	132.6	2,391	58
2010	2.4	5.8	141.7	2,417	59
2011	2.5	6.3	153.0	2,520	61
2012	2.6	6.9	178.6	2,654	69
2013	2.8	7.3	192.2	2,607	69
2014	2.9	7.6	204.4	2,621	70
2015	3.0	7.7	210.5	2,567	70
2016	3.2	7.9	227.3	2,469	71
2017	3.4	8.2	244.4	2,412	72
2018	3.6	8.3	254.5	2,306	71
2019	3.9	8.7	266	2,231	68
Total (increase ratio)^*^	85.71%	163.64%	170.60%	41.96%	45.71%

* The formula for calculating the percentage changes of all three indicators is “(data in 2019-data in 2007)/data in 2007 * 100%”.

**Figure 3:**
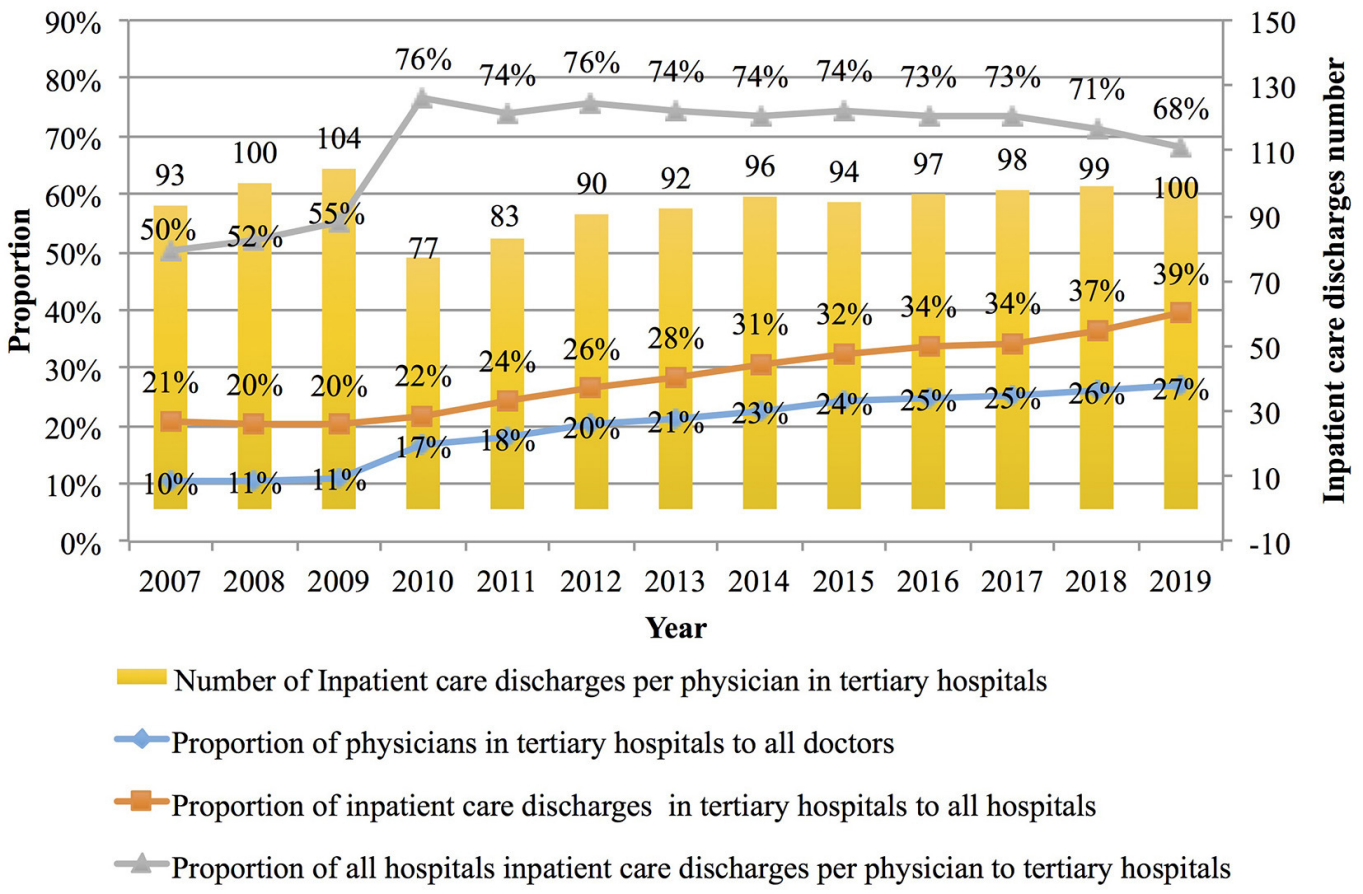
Average hospitalization workload of each doctor in Chinese hospitals from 2007 to 2019.

In China, there are significant differences in the workload of hospitals at different levels, and physicians in tertiary hospitals tend to have the highest workload. In 2019, the number of physicians in tertiary hospitals accounted for ~27% of physicians in all hospitals, but they undertook care for about 40% of inpatients (31); in other words, the number of hospitalized patients per capita under the responsibility of physicians across the country was only 68% of the average number of physicians affiliated with tertiary hospitals (see Figure 3 below for details).

**Figure 4:**
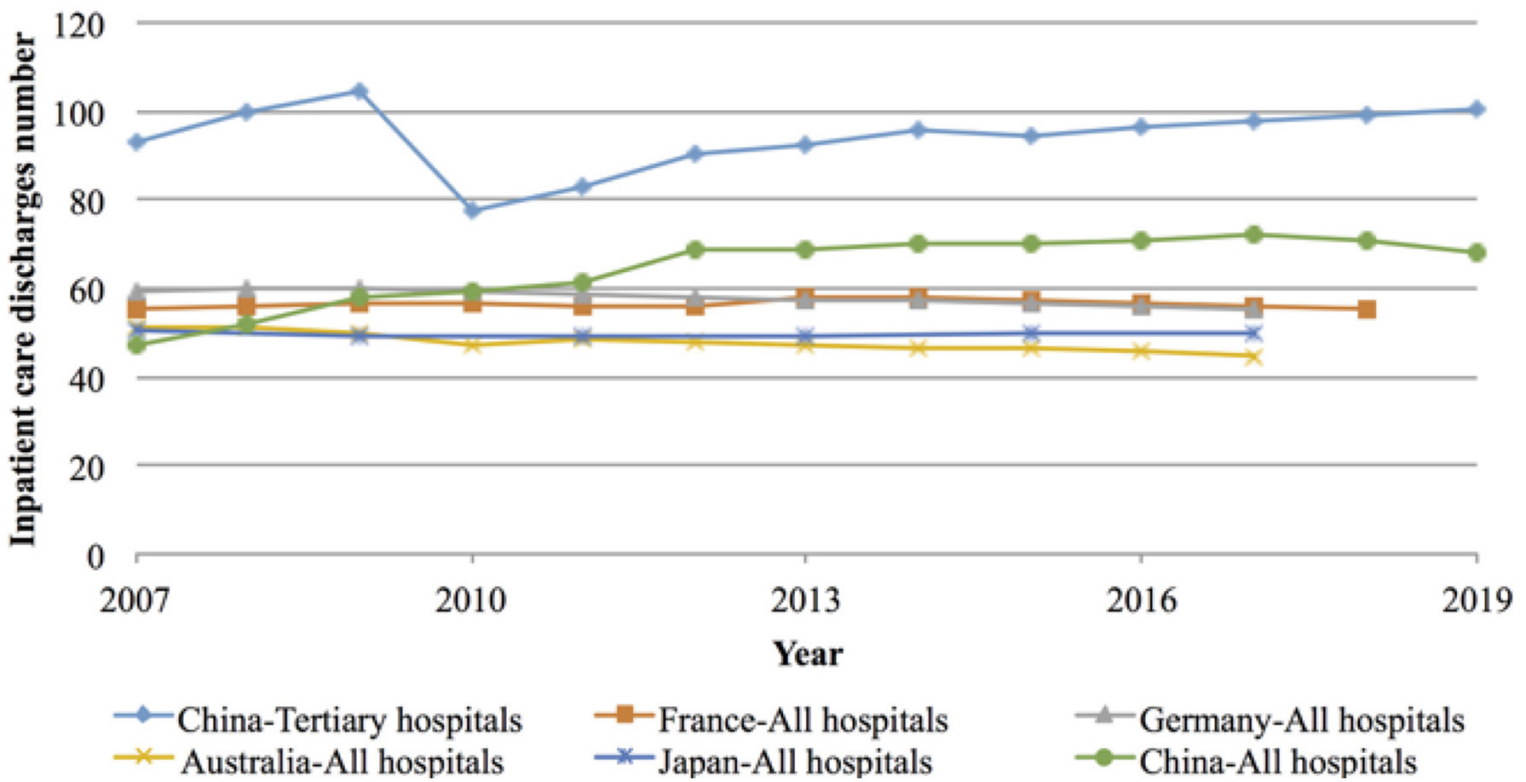
Workload of inpatients per doctor in Europe (France and Germany), Asia (Japan and China), and Oceania (Australia) from 2007 to 2019.

Compared with physicians in Europe (i.e., France and Germany), Asia (Japan), and Oceania (Australia), the workload of physicians in China is relatively large. In terms of inpatients received per physician, the workload of physicians in China exceeded that of their peers in France, Germany, Japan, and Australia from 2011 onwards, and this gap has gradually widened. By 2017, the number of inpatients received per physician in China, France, Germany, Japan, and Australia was 72, 56, 55, 50, and 45, respectively. In contrast, the number of inpatients received per physician in China's tertiary hospitals exceeded the sum of the average workload of their Australian and Japanese peers (98 vs. 45 + 50 inpatients) in 2017, and continued to increase until it reached 100 person-times per year by 2019 (see Figure 4 below for details).

Workload of inpatients per physician = Number of hospital discharges by diagnostic categories (32)/Number of professionally active physicians (33).

### Comparison and Correlation Analysis Based on the Workload and Abnormal Death Case Data

Long working hours and heavy workloads are considered risk factors for death by overwork, which is verified by the sample data. Although the number of outpatients received per physician in China decreased from 2,621 person-times/year in 2014 to 2,231 person-times/year in 2018, the number of inpatients received per physician reached a new high (increasing from 58 in 2008 to 68 in 2019), during which time the number in tertiary hospitals reached 100 persons/year. Pearson's product-moment correlation was used to analyze the correlation between the number of inpatients received per physician per year and the number of abnormal deaths. The strength of the correlation was assessed based on Cohen's criterion (34, 35), whereby correlations <0.30 were considered small; correlations between 0.30 and <0.50 were considered medium; and correlations of 0.50 or more were considered strong. There was a very high correlation between the number of abnormal deaths of physicians in China and the number of inpatients received per physician (*r* = 0.683, *P* = 0.01), whereas there was no statistically significant difference between the correlation results and the number of outpatients received per physician (*r* = 0.0455, *P* = 0.12). This might be related to the treatment or operation of inpatients, especially during night shifts and having long periods of being on call, which may have resulted in more occupational stress, anxiety, and burnout (36–38).

## Discussion

Through systematic information retrieval, this study collected 207 abnormal death events among physicians in China from 2007 to 2020, including overwork death (164), physician-patient disputes (36), and disputes among colleagues (7). The number of victims who were men was 5.5 times that of women, and victims were between the ages of 31–50 years at the time of death. The frequency of abnormal death events in tertiary hospitals was more than twice that of other hospitals. Victims were mainly working in surgery, anesthesiology, internal medicine, and orthopedics, which together accounted for 44% of all cases. Further analysis of the direct causes of overwork death showed that about one-third of doctors (39) died from cardiogenic diseases, including acute myocardial infarction and acute heart failure. The second direct cause was suicide (i.e., falling from a building), and the third was brain-derived diseases, including unexplained strokes and cerebral hemorrhages.

Furthermore, the per capita workload of physicians in China increased by about 42% between 2007 and 2019, far exceeding that of physicians in Europe (i.e., Germany and France), Asia (Japan), and Oceania (Australia) in 2017 (72 vs. 55, 56, 50, and 45, respectively); the per capita workload of physicians in tertiary hospitals also exceeded 100 person-times/year. Analysis showed a strong correlation between the number of abnormal deaths of physicians in China and the number of inpatients received per physician (*r* = 0.683, *P* = 0.01).

The most important contribution of this study may not be the data on abnormal death among physicians in China, but an awareness of the lack of data support for relevant scientific research and knowledge accumulation. First, information on the timing of such events is still not clear. Second, most public reports lack details of the events, which could support a more in-depth comparison and analysis. This suggests that it may be necessary to establish a systematic mechanism for medical institutions to report abnormal death events of physicians dominated by overwork death, including the direct cause of death, work status, living habits, and physical status of the victims before death, in order to reduce or even eliminate their occurrence, and to establish possible prevention and emergency response strategies.

### Characteristics and Trends of Abnormal Death Among Physicians in China

Due to the uneven distribution of medical resources, the inability to meet the rapidly growing demand for medical care, the increasing workload of physicians, and worsening social public opinion and medical care environments, the number of abnormal deaths of physicians in China has been increasing. The research results show that since China's second health reform in 2009, the government has made basic health insurance available to more people, particularly the poor and rural populations; however, this has come at a cost (16). From the perspective of fiscal expenditure, the average healthcare expenditure of the Chinese government accounted for only 5% of the total government spending in 2016, which is lower than the world average of 6.6% (40). From the perspective of physicians as a human resource, the number of physicians in China increased by 85.7% (1.5 million) between 2016 and 2020. However, from the perspective of the volume of healthcare services, the number of outpatients increased by 163.4% (5.4 billion person-times), while the number of discharged inpatients increased by 170.6% (168 million person-times), which greatly increased the workload of medical staff in China. Physicians who have fallen ill from constant overworking also face serious health problems, and thus may in turn be providing patients with lower quality services. Research by Kivimäki et al. revealed that overworking may increase the risk of various cardiogenic diseases, such as coronary heart disease, and brain-derived diseases, such as stroke and cerebral hemorrhage (41). Research by Takahashi also found that the direct causes of overwork death in Japan are cardiogenic diseases and brain-derived diseases (e.g., such as strokes) caused by stress from overworking (42). According to reports by the World Health Organization (WHO), compared with physicians working 35–40 h per week, physicians working long hours (≥55 h/week) have a 35% increased risk of having a stroke and a 17% increased risk of dying from ischemic heart diseases (43). In the sample used in this study, the cases of overwork death due to the abovementioned causes accounted for ~38.4% (63/164) of all cases. Therefore, management departments and physicians should pay attention to physicians' health conditions, as well as implement reasonable measures to balance work and rest.

This study also found that, except for 2018, the number of abnormal deaths of physicians in China generally increased from 2007 to 2020. The model predicts a potential of 45 cases/year by 2025 (*R*^2^ = 0.741). The frequent incidence of overwork death of physicians might be attributable to the social and psychological pressure caused by long working durations, high intensity work, and poor medical care environments. According to a survey conducted by the Chinese Medical Doctor Association in 2017, the average weekly working hours of physicians in secondary/tertiary hospitals in Chinese cities was at least 52 h, which is far higher than the statutory 40 h of work per week in China; moreover, it was reported that 37% of physicians worked more than 60 h per week, 47% of physicians rested only 1 day per week, and 75% of physicians did not have paid leave (44). Furthermore, considering the number of discharged inpatients as an example, from 2007 to 2020, we found that the number of inpatients received per physician in China far exceeded those in Germany, France, Japan, and Australia (72 vs. 55, 56, 50, and 45, respectively); this number also exceeded 100 person-times/year among physicians in tertiary hospitals, which was equivalent to the sum of the average workload of their peers in Germany and Australia. Furthermore, the correlation between per capita workload and the number of abnormal deaths was very high (*r* = 0.683, *P* = 0.01). Moreover, China's three-tier diagnosis and treatment system is not as complete and comprehensive as that of Western countries. Both mildly and critically ill patients can directly visit tertiary hospitals to seek better medical resources. This means that tertiary hospitals, which account for only 11.6% of all hospitals, are responsible for about 39.4% of the inpatient workload, with a per capita workload exceeding the national average of 38.9%. At the same time, high-quality medical resources are mainly distributed in economically developed regions in the central and eastern regions, as well as municipalities directly under the central government. Our study found that the GDP of the provinces and cities with frequent abnormal deaths of physicians were all in the top 1/3 of the national rankings. In addition, overworking among physicians may aggravate the communication barriers between physicians and patients and intensify their contradictions, and may lead to physical violence against physicians or even death by homicide (45). A survey on physician-patient relationships conducted in 30 hospitals showed that only 10% of patients were still willing to trust physicians (46). In 2010, there were a total of 17,000 cases of patients or their families physically beating physicians in China, an increase of more than 7,000 cases compared with 2005; in 2013, such events rose to more than 70,000 cases (10). This work pressure and psychological burden may further aggravate the current situation of overwork death among physicians in China. Fortunately, the Chinese government has initiated a series of efforts and attempts to address this issue. For example, in August 2015, medical care violence was included in the criminal jurisdiction, and beginning in 2018, August 19 has been designated as “Chinese Doctors' Day” (47). In June 2020, the first comprehensive law in the field of healthcare in China, the *Law of the People's Republic of China on the Promotion of Basic Medical and Health Care*, was officially enforced to further protect the rights and interests of medical professionals (48). The *Measures for Protection of the Rights and Interests of Healthcare Professionals of Shanghai City*, which was piloted in Shanghai in December 2020, includes mandatory rest for overworked medical professionals, the inclusion of medical care violence events in leadership assessment, the adoption of haven protection measures for safety of medical professionals, and the cancellation of medical treatment eligibility for those who have injured or killed medical professionals. The effects remain to be seen (49).

### Adverse Effects of Abnormal Death Among Physicians in China

Under continuous and significant professional, social, and living pressures, the number of physicians has decreased. The education and cultivation of medical students could have contributed more to medical reform, but the effect has not been satisfactory. From 2005 to 2014, 4,727,977 medical students were trained in the Chinese mainland. However, during this period, the number of physicians increased by only 752,233 (50). According to the demographic data of physicians over the past decade, the number of physicians between the ages of 25–34 years have declined from 31.13 to 22.6%, while the proportion of physicians over the age of 60 rose from 2.5 to 11.6%. The number of physicians in rural areas has declined to more than 500,000 (50). We believe that this is caused by a multitude of factors. First, the professional value of physicians was seriously underestimated. Current public hospitals can only receive very limited financial assistance from the government; thus, they must be self-financing (13). The current calculation method for income per hospital is based on the number of examinations prescribed, medication orders issued, and operations completed, rather than the quality of diagnosis and treatment, which may produce an economic incentive for medical care (51). To minimize inappropriate conflicts of interest, the Chinese government has enacted laws to prevent physicians from obtaining additional illegal income from drugs and medical consumables (39, 52). Additionally, many Chinese patients believe that physicians and hospitals conspire to add a lot of unnecessary medical expenses and claim that physicians lack dedication and professional skills (53). Therefore, the income of physicians is not high. Many doctors need to maintain a delicate balance between professional ethics and a decent life, which also makes many medical students reluctant to continue in the industry. Second, medical schools have a heavy learning burden and require many years of education (i.e., Chinese medical students are required to undergo 5 years of medical education and 3 years of standardized resident training to become formal doctors; to obtain a doctoral degree, an additional 6 years is required) (54). The long study cycle and relatively low income have caused more students to abandon the study of medicine and have led to reduced student enrollment. Moreover, there is academic and teaching pressure for physicians working in tertiary hospitals. They are required to publish research and finish teaching in order to be promoted and recognized by their peers. In China, patient satisfaction is based on the recognition of physicians' ability, and physicians' reputations largely depend on their research results (13), especially high-impact academic papers (55). This makes it necessary for physicians to spend their time conducting scientific research. Under this high load working environment, it is not surprising that the proportion of abnormal deaths among physicians in tertiary hospitals is high, and this pressure is likely to be transmitted to the vast number of medical students who are undergoing clinical studies in tertiary hospitals.

### Possible Causes and Proposed Response Measures

There are four possible causes of most abnormal deaths among physicians in China: first, the long and intensive nature of their work; second, the unreasonable allocation of medical resources (i.e., tertiary hospitals are overcrowded, with an imbalance between supply and demand, so that a large proportion of abnormal deaths occur in tertiary hospitals); third, mental stress, including tension between physicians and patients, as well as high pressure to conduct scientific and academic research; fourth, human resource reserves are insufficient. On the one hand, we suggest limiting the ultra-large-scale expansion of hospitals, increasing the number of physicians in large hospitals, and reducing their workload. On the other hand, by reasonable triage of patients and vigorously developing community hospitals, the burden of tertiary hospitals can be reduced; the remuneration package of physicians, including medical school graduates, can also be improved to attract medical school graduates to return to their profession. In addition, we should reform the existing health management system and improve the integrated health improvement system of tertiary and community hospitals. Such measures can be supplemented by relevant laws and regulations (i.e., the international mode of settling medical disputes by third-party intervention and the implementation of a mandatory leave system) to solve the high-pressure problems that physicians are experiencing. In this way, we can also coordinate the relationship between scientific research and clinical practice to find a more fair and effective way of professional title accreditation and performance evaluation. Furthermore, hospitals can set up special physician lounges, improve the shift system with compensation for overtime, and organize a system for physicians to undergo physical examinations on a regular basis.

### Smart Hospital Technology Optimizes Service Supply, Improves Medical Experience, and Balances the Workload of Physicians

The sharpness of physician-patient contradictions is related to the imbalance in the supply of medical services. HIT-based smart hospitals can partially balance the workload of physicians, optimize the supply of medical services, and improve patient experience. In China, the government began to gradually propose and improve the concept and policy of the “smart hospital” in 2018, to the best possible, to reduce the burden of medical staff on indirect medical work. From the perspective of physicians, “smart medical care” would help the reconstruction and optimization of the existing workflow. As an important technical means, HIT has the potential to improve service quality, improve service efficiency, ensure service safety, and reduce the cognitive and mental load of physicians at work (56). According to CHIMA Annual Surveys, the average electronic medical records adoption rate in sampled hospitals in China increased 3.6 times from 2007 to 2018, peaking at 85.3% (30). Based on the experience of foreign counterparts, with forward-looking top-level policy arrangements, good HIT software design and implementation, longer HIT clinical use time and adequate coverage, as well as necessary policy guidance, HIT can fulfill its potential promise to the medical industry and medical services by reducing the workload of physicians to a certain extent (57–59). From the perspective of patients, through “smart services,” indirect medical services such as registration, charging, examination, inspection, admission, and discharge are streamlined for patients. For example, through mHealth, telemedicine and health kiosks technologies, patient self-service functions such as appointment registration, various report queries, off-site admission, and discharge payment can be developed (60), providing patients with more convenient, simple, and considerate medical services, improving the patient's medical experience, and reducing the effort of medical staff. From the perspective of hospital management, through “smart management,” non-patient-oriented transactional operations are reduced for physicians, including the operation of various equipment and facilities, drug consumables application, scientific research, and teaching management, and physicians are returned to patients.

## Limitations

This study also has some limitations. First of all, this study is a retrospective mixed study based on public news and literature reports, and the included data samples may only be part of the actual events. Second, not all research results obtained in China can be extended to other countries. In particular, China's medical and health system may be very different from the international mainstream. Therefore, caution is required when using the findings of this study in medical settings in other countries. Then, the purpose of this research is to provide information for decision-makers to formulate better policies to prevent and control physicians' abnormal deaths and to increase the awareness of self-awareness and self-protection among doctors, without further processing of data.

## Summary

The overwork of physicians is a worldwide problem. However, the huge workload and mental stress in the Chinese medical environment, strained human resources, severe violent tendencies toward medical care, and the deterioration of doctor-patient relationship and trust have exacerbated the professional burnout of Chinese doctors. This study attempts to describe the common pattern of abnormal deaths among Chinese physicians, and proposes some suggestions including smart hospital technologies for improvement as the first step in establishing a long-term mechanism to protect medical staff. Fortunately, the Chinese government has also promulgated a series of laws and regulations to improve the medical environment and accelerate the reform of the hierarchical medical system, but these measures may not be enough. China's medical reform has a long way to go, and we must move forward.

## Data Availability Statement

The original contributions presented in the study are included in the article/Supplementary Material, further inquiries can be directed to the corresponding author/s.

## Author Contributions

JLe formed the conception and study design and made significant revisions. JLi, YH, XZ, TW, HQ, and LF did the literature review and undertook data acquisition and data analysis. JLi drafted the manuscript. MN, DS, and JX supervised the review method and interpretation of data and supplied valuable improvement suggestions. The work presented here was carried out in collaboration among all authors.

## Funding

This work was supported by the National Natural Science Foundation of China (Grant Nos. 81771937 and 81871455), Zhejiang Provincial Natural Science Foundation of China (Grant No. LZ18F020002), and partly supported by PKU-Baidu Fund (project of intelligent auxiliary diagnosis using medical images).

## Conflict of Interest

The authors declare that the research was conducted in the absence of any commercial or financial relationships that could be construed as a potential conflict of interest.

## Publisher's Note

All claims expressed in this article are solely those of the authors and do not necessarily represent those of their affiliated organizations, or those of the publisher, the editors and the reviewers. Any product that may be evaluated in this article, or claim that may be made by its manufacturer, is not guaranteed or endorsed by the publisher.
