# Supplementary material for: A Preliminary Study on the Abnormal Deaths and Work Burden of Chinese Physicians: A Mixed Method Analysis and Implications for Smart Hospital Management
Source: Front Public Health. 2022 Jan 4;9:803089. doi: 10.3389/fpubh.2021.803089 (PMC8764251; doi:10.3389/fpubh.2021.803089)
Supplement: Supplementary file 1 [file Data_Sheet_1.DOCX]

Annex 1

Working status and working environment of Chinese doctors

The health and living conditions of doctors are of vital importance to medical treatment, but excessive work pressure can damage the health of doctors and even lead to death from overwork. Overwork(Karoshi), comes from Japanese, is an occupational sudden death, the first recorded death from overwork occurred in Japan in 1969 [1]。Japan National Institute of Health and Medical Sciences(Institute of Public Health) Professor Uehata of Japan National Institute of Health and Medical Sciences(Institute of Public Health) reported five work patterns that may lead to death from overwork, including: all-weather high-intensity work; extremely long working hours or long-term day and night inversions; uninterrupted continuous work without rest; extremely heavy physical work; high mental stress work [2]. Unfortunately, some of them have become the norm for some Chinese doctors. Due to space, please refer to Annex 1 for the description of the working status of Chinese doctors. First of all, the work pressure of doctors is imbalanced in the context of the imbalance between the supply and demand of medical resources. Compared with countries with complete hierarchical diagnosis and treatment systems, Chinese patients can freely choose outpatient hospitals, resulting in "overcrowded" tertiary hospitals with higher medical standards. In 2019, the outpatient workload of tertiary hospitals, which accounted for only 11.6% of all hospitals, exceeded that of other hospitals combined, reaching 51.9% [3]. Some doctors work very hard, doctors even dare not drink water for fear of going to the toilet when receiving patients in outpatient clinics [4]. In 2017, YuFang Hospital Management Center conducted a questionnaire survey of 4617 medical staff across the country. More than 72.06% of the doctors' respondents said that they were tired or very tired after get off work, and more than 56.29% of the respondents said they were working in the hospital. The total time for breakfast and lunch does not exceed 10 minutes [5]. Secondly, the work of Chinese doctors is not only intense, but also long-term, and night shifts are often required. The average working time of doctors is at least 52 hours per week. Among them, 26-35 years old have the longest working hours per week, which is 53 hours, which is far higher than the legal requirement of 40 hours per week in China [6]. Then, the implementation of the vacation system among doctors is also very poor. According to the survey results of the “White Paper on the Occupational Status of Chinese Doctors” conducted by the Chinese Medical Doctor Association in 2017 for 146,200 doctors, only 24% of the respondents indicated that they could enjoy two-day breaks, and even 23.6% of the respondents did not enjoy paid vacation [6]. In addition, the psychological pressure of doctors is generally greater. The "White Paper on the Professional Status of Chinese Doctors" also uses psychological exhaustion indicators to evaluate the degree of psychological damage caused by work pressure. It is found that the overall psychological exhaustion level of the doctor group is significantly higher than the corporate employee reference group, among them, 51.3% of respondents with mental exhaustion level above 6 (severe), anesthesiologists even as high as 69% [6].

Chinese doctors frequently receive abnormal death threats including overwork/sudden death and homicide, which are not only medical problems but also social problems. High-intensity, high-risk, and high-response work causes doctors to accumulate excessive fatigue, physical and emotional exhaustion. On the one hand, deaths from overwork/sudden deaths frequently occur in the doctor community [7,8]. On the other hand, this will also increase the risk of adverse medical events and intensify the conflict between doctors and patients. In 2010, there were 17,000 cases of beatings of doctors by patients or their families in China, an increase of more than 7,000 from 2005. In 2013, such incidents had risen to more than 70,000 [9]. According to a survey of 2,617 medical staff covering 30 provincial administrative regions in China, about 76.2% of medical staff have experienced at least one WPV(Workplace violence)experience in the past year[10], even 28% of victims suffer from post-traumatic stress disorder [11]. In recent years, there have been many reports about doctors being severely violent or even killed by patients or their families [12-14]. In addition, in this continuous negative public opinion and environment, not only is the number of medical students applying for the exams insufficient, for example, in Hebei Province in 2015, the admission score of veterinarians for college entrance examinations exceeded the online percentage of admissions for medical majors [15], but also it has accelerated the loss of existing doctors and further aggravated the shortage of human resources. In some hospitals, the vacancy rate was even as high as 20.7% [16].

References:

[1] Wikipedia, "Karoshi [Online]", Available: https://en.wikipedia.org/wiki/Karoshi.

[2] Uehata T., "Long working hours and occupational stress-related cardiovascular attacks among middle-aged workers in Japan", J Hum Ergol (Tokyo), vol. 20, no. 2, pp.147-153, 1991.

[3] National Health Commission of the People's Republic of China, 2020 China Health Statistics Yearbook, Peking Union Medical College Press, Beijing, 2020.

[4] Wu H., Fu S., "Afraid of going to the toilet and dare not drink water, 17 medical staff are spinning around 24 hours [Online]", Available: https://rmh.pdnews.cn/Pc/ArtInfoApi/article?id=11157750.

[5] Fang Q., "Heavy report: Doctors' physical and mental health is worrying [Online]", Available: https://www.cn-healthcare.com/articlewm/20180112/content-1021621.html.

[6] Chinese Medical Doctor Association, "White paper on the practice status of Chinese physicians", Chinese Medical Doctor Association, Beijing, 2018.

[7] Song X.N., Shen J. et al., "Sudden Deaths Among Chinese Physicians", Chin Med J (Engl), vol. 128, no. 23, pp.3251-3253, 2015.

[8] Gao X., Xu Y., "Overwork death among doctors a challenging issue in China", International Journal Of Cardiology, vol. 289, pp.152, 2019.

[9] Zhang L., Stone T.E. et al., "Understanding the rise of Yinao in China: A commentary on the little known phenomenon of healthcare violence", Nurs Health Sci, vol. 19, no. 2, pp.183-187, 2017.

[10] Sun T., Gao L. et al., "Workplace violence, psychological stress, sleep quality and subjective health in Chinese doctors: a large cross-sectional study", BMJ Open, vol. 7, no. 12, pp.e017182, 2017.

[11] Shi L., Wang L. et al., "Prevalence and correlates of symptoms of post-traumatic stress disorder among Chinese healthcare workers exposed to physical violence: a cross-sectional study", BMJ Open, vol. 7, no. 7, pp.e016810, 2017.

[12] The Lancet, "Chinese doctors are under threat", The Lancet, vol. 376, no. 9742, pp.657, 2010.

[13] "The killing of the doctor in Wenling city, Zhejiang provide, China [Online]", Available: https://zh.wikipedia.org/wiki/%E6%B5%99%E6%B1%9F%E6%B8%A9%E5%B2%AD%E8%A2%AD%E5%8C%BB%E4%BA%8B%E4%BB%B6.

[14] The L., "Protecting Chinese doctors", Lancet, vol. 395, no. 10218, pp.90, 2020.

[15] Xiao W., "Medical professional scores are not as good as veterinarians [Online]", Available: <http://hainan.ifeng.com/news/fengguan/detail_2015_07/02/4068359_0.shtml>.

[16] Shen J., Chen N. et al., "The hospital director is under great pressure! Serious loss of key doctors in the county [Online]", Available: https://www.cn-healthcare.com/article/20191016/content-524820.html.
