# Supplementary material for: A Preliminary Study on the Abnormal Deaths and Work Burden of Chinese Physicians: A Mixed Method Analysis and Implications for Smart Hospital Management
Source: Front Public Health. 2022 Jan 4;9:803089. doi: 10.3389/fpubh.2021.803089 (PMC8764251; doi:10.3389/fpubh.2021.803089)
Supplement: Supplementary file 2 [file Data_Sheet_2.DOCX]

Annex 2

Search terms

1. Search engine (Baidu、Google and Sogou)

Chinese search terms: (医生 或 过劳死 或 医生猝死 或 杀医 或 暴力伤医 或 护士杀医)

English translation: (Doctors OR Overwork OR Doctor's sudden death OR Kill doctor OR Violent Wound Doctor OR Nurse killing doctor)

1. Chinese scientific literature database search term

Chinese theme = （杀医 或 暴力伤医 或 护士杀医生 或 医生过劳死 或 医生猝死）

English translation: (Kill doctor OR Violent Wound Doctor OR Nurse kills doctor OR The doctor is overworked OR Doctor's sudden death)

1. English scientific literature database search terms (refer to the previous article on the death of Chinese doctors from overwork)
   1. PubMed

(‘Chinese doctors’ or ‘Chinese physicians’) and (‘Karoshi’ or ‘sudden death’ or ‘overwork death’ or ‘injured death’ or ‘threat death’ or ‘violence death’) AND Language: (English) AND Document type: (Article OR Review)

- 1. WOS

(TS=(china) AND TS=(doctor OR physician) AND TS=(karoshi OR sudden death OR overwork death OR injured death OR threat death OR violence death)) AND Language: (English) AND Document type: (Article OR Review)
